# Experience with PCR Testing for Enteric Bacteria and Viruses of Emergency Department Patients with Acute Gastroenteritis: Are There Implications for the Early Treatment of *Clostridioides difficile* Infection?

**DOI:** 10.3390/antibiotics13030243

**Published:** 2024-03-06

**Authors:** Andreas Iffland, Maria Zechel, Jan-Christoph Lewejohann, Birgit Edel, Stefan Hagel, Michael Hartmann, Bettina Löffler, Jürgen Rödel

**Affiliations:** 1 Hospital Pharmacy, Jena University Hospital, Friedrich Schiller University, 07747 Jena, Germany; andreas.iffland@med.uni-jena.de (A.I.); michael.hartmann@med.uni-jena.de (M.H.); 2 Institute of Infectious Diseases and Infection Control, Jena University Hospital, Friedrich Schiller University, 07747 Jena, Germanystefan.hagel@med.uni-jena.de (S.H.); 3 Department of Emergency Medicine, Jena University Hospital, Friedrich Schiller University, 07747 Jena, Germany; jan-christoph.lewejohann@med.uni-jena.de; 4 Institute of Medical Microbiology, Jena University Hospital, Friedrich Schiller University, 07747 Jena, Germany; birgit.edel@med.uni-jena.de (B.E.); bettina.loeffler@med.uni-jena.de (B.L.)

**Keywords:** acute gastroenteritis, emergency department, PCR, *Clostridioides difficile*

## Abstract

Early identification of acute gastroenteritis (AGE) pathogens via PCR may improve the management of patients presenting to the emergency department (ED). In this study, we evaluated the implementation of a testing algorithm for ED patients with AGE using the BD MAX automated PCR system. Data from 133 patients were analyzed. A total of 56 patients (42%) tested positive via PCR for at least one bacterial or viral pathogen. The median time to report PCR results was 6.17 h compared to 57.28 h for culture results for bacterial pathogens. The most common pathogen was *Clostridioides difficile* (*n* = 20, 15%). In total, 14 of the 20 *C. difficile*-positive patients were aged >65 years and 17 of the 20 patients (85%) were diagnosed with a clinically relevant infection based on typical symptoms and laboratory values. They received antibiotics, mostly oral vancomycin, starting a median of 11.37 h after ED admission. The introduction of PCR for the diagnosis of AGE infection in patients presenting to the ED may have the greatest impact on the rapid identification of *C. difficile* and the timely administration of antibiotics if necessary.

## 1. Introduction

Multiplex PCR is being increasingly used for the diagnosis of acute gastroenteritis (AGE), not only to increase diagnostic sensitivity but also to provide timely diagnosis compared to bacterial culture and conventional stepwise diagnostics [1,2]. However, PCR cannot replace the isolation of bacterial pathogens by culture, which is required for serotyping and accurate characterisation of different pathotypes, e.g., Shiga toxin-producing *E. coli* (STEC) and *Salmonella*. PCR causes additional laboratory costs, and the clinical impact in terms of specific therapy is limited [1]. In the outpatient setting, testing for viruses is not indicated because the results have no impact on the treatment of the patient [3]. However, identification of the bacterial pathogen may be helpful in deciding whether to use an antibiotic, for example, in cases of Shigellosis, prolonged *Campylobacter* infection, severe symptoms, or underlying chronic disease [3,4]. In the hospital setting, PCR testing for viruses and bacteria could also improve patient hygiene management. However, broad-spectrum multiplex PCR testing is not recommended for patients who have been hospitalised for more than 48 h because infectious hospital-associated AGE is associated with a small spectrum of pathogens, mainly norovirus and *Clostridioides difficile* [3].

The emergency department (ED) is a critical interface for timely diagnostics in a tertiary hospital. Immediate testing of patients presenting with AGE in the ED may help to improve patient management, as they need to be isolated when admitted to a ward and this may be important for timely therapeutic decisions. Many patients presenting to the ED with severe illness belong to vulnerable populations, such as the elderly or immunocompromised. For these patient groups, broad panel testing for AGE pathogens may be most relevant. This also includes children, who are known to have an increased incidence of AGE [1,5]. As there is a large overlap in clinical symptoms caused by different pathogens, it is challenging to differentiate patients for more individualized testing to avoid delays in diagnostic reporting and to simplify the workflow for the emergency department staff. This problem also applies to testing for *C. difficile*, which can cause mild disease as well as severe pseudomembranous colitis [6].

Here we report the results of an observational study on the testing of ED patients with AGE using the enteric viral panel (EVP), enteric bacterial panel (EBP), and Cdiff PCR assays of the automated BD MAX^™^ system (BD, Heidelberg, Germany). All assays were performed in parallel from the same sample, including the PCR assay as a one-step diagnostic strategy for *C. difficile* infection (CDI).

## 2. Results

### 2.1. BD MAX PCR Testing

During the study period, a total of 133 patients admitted to the ED and presenting with diarrhea were tested with the BD MAX^™^ enteric assays. Data analysis included both adults and children <18 years of age. The median age of the patients was 60 years (IQR 10–80). Of the patients, 68 were female (51.13%) and 65 were male (48.87%). Sixty-two patients were >65 years of age (46.62%). Overall, 56 patients (42.1%) tested positive for at least one pathogen (Table 1). For pathogens that were tested using both PCR and culture in 19 out of 21 PCR-positive samples (90.48%), the corresponding pathogen could be isolated (*Campylobacter* spp. 9/10: *C. jejuni* (*n* = 8) and *C. coli* (*n* = 1); *Salmonella* spp. 8/8: *S. Enteritidis* (*n* = 4), *S. Typhimurium* (*n* = 2), *S. Goldcoast* (*n* = 1), and *S. Infantis* (*n* = 1); STEC 1/2: STX-1-positive *E. coli*; *Shigella* spp. 1/1: *S. sonnei*). The median time from the laboratory request to the reporting of PCR results was 6.17 h (IQR 4.07–15.25) compared to a median time of 57.28 h (IQR 44.25–64.87) for the reporting of bacterial culture results (*p* < 0.001, Mann–Whitney U test). The median length of stay in the ED before transfer to a ward was 5.65 h (IQR 3.37–8.83).

### 2.2. Hygiene Management

Our hospital hygiene management requires the isolation of patients with diarrhea until a completely negative diagnostic result has been obtained. There were two cases of patients in whom a pathogen not included in the PCR panels used here was subsequently identified. In one of these cases, *Yersinia enterocolitica* was isolated from the same sample and another patient tested positive for *Cryptosporidium parvum* in a second sample. Of note, additional BD Max PCR panels covering both pathogens are available but were not used for the initial testing due to the low incidence of these pathogens to reduce diagnostic costs. In total, 85 out of 133 patients were admitted to a hospital ward. While there was no significant difference in the length of hospital stay between PCR-negative and PCR-positive patients, the latter spent more time in single-room isolation (Table 2). This was because 24% of the hospitalized patients with a negative PCR result (12/50) but only 2.9% of patients with a positive PCR result (1/35) were initially not correctly isolated after transfer to a ward (*p* = 0.027, Fisher’s exact test).

### 2.3. C. difficile

The most common pathogen detected was *C. difficile*, with 20 cases out of 133 patients (15%), and as expected, the majority of cases were in the older patient group (Table 3). A total of 18 out of 20 patients (90%) were hospitalized. Therefore, further data analysis on antibiotic treatment decisions focused on these cases. According to international guidelines, the diagnosis of CDI was based on the criteria of a positive laboratory test in the presence of diarrhea and/or pseudomembranous colitis, taking into account additional parameters such as fever, elevated white blood cell (WBC) count, and a 50% increase in serum creatinine level. In three patients (15%), including two children, the positive PCR result was not clinically relevant due to the absence of characteristic symptoms or symptoms of other causes (Table 3). Eleven patients (55%) were diagnosed with CDI for the first time. PCR Ct values showed a wide variation in cases of clinical relevance and did not correlate with WBC or creatinine laboratory parameters (Table 3). Specific antibiotic treatment was preferentially initiated with vancomycin, as recommended (Table 3). For cases for which the time of initiation of antibiotic therapy was available in the hospital information system, the median time from ED presentation to antibiotic administration was 11.37 h (IQR 7.22–15.25) and the median time to report a positive *C. difficile toxB* PCR result was 8.9 h (IQR 4.83–13.75). It should be noted that, in several cases, vancomycin was given before the PCR result was obtained. One of the hospitalized *C. difficile*-positive patients was not correctly isolated as recommended. For the other patients, the median time of isolation was 182.33 h (IQR 140.44–235) compared to a median hospital length of stay of 193.98 h (IQR 143.06–264.16). Fulminant cases of CDI were not observed in this study.

## 3. Discussion

The implementation of PCR into the diagnostic workflow of AGE may result in a benefit for optimizing patient management [1,4,7]. In this study, the value of PCR for testing ED patients was examined. For bacterial pathogens that are conventionally diagnosed by culture, the detection rate was slightly increased and the main **advantage** of PCR testing was a shorter time to report positive test **results**. As expected, the most commonly detected enteric virus was norovirus, an important pathogen often associated with transmission between patients in close contact in hospitals leading to nosocomial infections [3]. Timely diagnosis of AGE may have a therapeutic benefit by both reducing unnecessarily **prescribed** antibiotics and targeting a more specific therapy when needed but the impact of rapid multiplex PCR on patient hygiene management should not be overestimated [1,3,8]. As PCR cannot exclude all pathogens, patients with AGE but a negative screening PCR result are isolated upon admission to a ward until all diagnostic test results are available or until 48 h after suspending diarrhea. However, errors in hygiene management, as also observed in this study, may occur, and it can be argued that staff will be better sensitized to isolate the patient for whom a positive PCR test is reported in a timely manner.

The most common pathogen detected in ED patients was *C. difficile*, which, as expected, was detected in higher numbers in the older patient group [6]. We included PCR testing for *C. difficile* as a one-step diagnostic in the workflow. This did not strictly follow the preferred workflow in the diagnostic guidelines of the *European Society of Clinical Microbiology and Infectious Diseases* (ESCMID) and the *Infectious* Diseases Society of America (IDSA) which recommend a two-step diagnostic algorithm consisting of a sensitive screening test and a less sensitive confirmatory *C. difficile* toxin assay [9,10]. The use of a PCR test alone is only a weak recommendation when there are specific institutional criteria for the submission of patient stool samples [10]. Confirmatory testing with a second assay is suggested to reduce the number of clinically irrelevant results, thereby reducing overdiagnosis of CDI [11]. *C. difficile* can cause only colonization or disease with a wide variety of symptoms, ranging from diarrhea, vomiting, fever, and abdominal pain to severe pseudomembranous colitis and even septic shock [6]. The use of PCR as a stand-alone test has not been defined as an optimal approach to differentiate between colonization and infection [9]. On the other hand, there are studies in which the use of one-step PCR testing results in more patients being accurately diagnosed and fewer deaths, indicating that suboptimal analytical sensitivity in two-step algorithms may miss cases that should be treated [12,13,14]. It has been shown that about 70% of PCR-positive but toxin antigen-negative patients were found to have probable or possible CDI [13]. It is therefore not surprising that the guidelines also state that a positive PCR screening test in combination with a negative toxin antigen test needs to be clinically evaluated. However, this strategy may lead to excessive repeat testing. In this study, the decision was made to include *C. difficile* in the PCR panel as a one-step testing strategy in order to reduce the workload and spare time. *C. difficile* testing is not routinely recommended for children as standard diagnostics but was included in this study in order to simplify the workflow [10,15]. Although low PCR Ct values have been reported to be associated with clinically relevant infections, no Ct cut-off values can be defined to distinguish colonization from infection [16,17]. In addition to clinical symptoms, elevated WBC count and serum creatinine levels may be considered in the diagnosis of CDI as they may indicate the development of severe infection [18]. In this study, there was no correlation between low PCR Ct values and both WBC count and serum creatinine, and all parameters should be used together to assess clinical relevance.

The ED is critical for the early identification of CDI in patients presenting with AGE [18,19]. Reassessment of admitted patients in the following days may not be the optimal method for CDI diagnosis and the timely initiation of antibiotic treatment to prevent the development of severe or fulminant disease [18]. As shown here, the majority of ED patients with a positive PCR test result were diagnosed with a clinically relevant infection that required treatment. Based on the information available in the patient records, approximately 69% of the patients had no recent history of CDI and the result was interpreted as a first diagnosis. The most commonly used antibiotic was oral vancomycin [20]. In our hospital-specific guidelines, fidaxomicin is preferentially recommended for the treatment of patients at high risk of recurrent infection [20].

In conclusion, this study shows that the implementation of PCR testing for ED patients with AGE has the greatest impact on the early detection of CDI in the elderly patient population. As CDI can result in severe disease associated with high mortality and the risk of readmission in patients with underlying disease or high age, *C. difficile* diagnosis based on PCR testing alone in the context of clinical symptoms is acceptable for therapeutic decisions. Timely reporting of positive PCR results may also increase attention to basic hygiene measures like hand hygiene and pathogen-specific measures such as isolation and personal protective equipment.

## 4. Materials and Methods

### 4.1. PCR Testing and Microbiology Diagnostic Workflows

The samples were unpreserved stool samples collected from patients with acute gastroenteritis who presented to the ED of the Jena University Hospital between July 2019 and January 2022. Samples were transported to the microbiology laboratory via the central pneumatic tube system and immediately tested with the BD MAX^®^ EBP, EVP, and Cdiff assays during the normal working day between 7 a.m. and 5 p.m. Samples arriving after 5 p.m. were analyzed in the morning of the next day. Test results were reported by telephone to the ED as soon as possible between 7 a.m. and 8 p.m.

The BD MAX^™^ EBP detects *Salmonella* spp., *Shigella* spp./EIEC, *Campylobacter jejuni* and *C. coli*, and stx (indicative of STEC) without distinguishing between stx1 and stx2. The BD MAX^™^ EVP includes targets for norovirus (genogroups I and II), rotavirus A, adenovirus (types F40 and F41), sapovirus (genogroups I, II, IV, and V), and human astrovirus. The BD MAX^™^ Cdiff assay targets the toxin B gene (tcdB) of *C. difficile*.

For bacterial cultures, samples were inoculated on Hektoen agar, salmonella–shigella agar, Butzler agar (Thermo Fisher Scientific, Wesel, Germany), a selenite broth (BD) at 37 °C, and a second selenite broth at 30 °C. Cultures were incubated for two days with daily visits for the growth of suspect colonies. Brilliance *E. coli*/coliform chromogenic agar (Thermo Fischer Scientific, Wesel, Germany) and GN broth (BD) were used for the isolation of STEC. Diagnosis on *Y. enterocolitica*, which is not included as a target in the BD MAX^™^ EBP, was performed through inoculation on CIN (Thermo Fisher Scientific) agar incubated at 30 °C. The following methods were used to identify bacterial pathogens in the culture: Vitek MS (bioMeriéux, Nürtingen, Germany), eazyplex^®^ SalmoTyper LAMP assay (Amplex Diagnostics, Gars-Bahnhof, Germany), and seroagglutination (Sifin Diagnostics, Berlin, Germany) for *Salmonella* spp.; API E (bioMeriéux) and seroagglutination for *Shigella* spp.; eazyplex^®^ EHEC complete LAMP assay (Amplex Diagnostics) for STEC; Vitek MS for *Campylobacter* spp.; and Vitek MS and seroagglutination for *Y. entercolitica*.

All assays were performed according to the manufacturer’s protocols.

### 4.2. Data Analysis

The turnaround times of PCR and culture microbiology diagnosis were calculated using the times recorded in the laboratory information system when sample testing was requested, and the results were reported to the ED. Laboratory data (WBC and creatinine) of patients who tested positive for *C. difficile* were obtained from the laboratory information systems. Medical records were reviewed to identify patients’ symptoms and co-morbidities, to determine when antibiotic therapy was changed or started after admission, and to calculate the duration of single-room isolation. Fisher’s exact and Mann–Whitney U tests for statistical analysis were performed using the Statistics Kingdom online tool (www.statskingdom.com).

## Figures and Tables

**Table 1:** BD MAX PCR results.

Pathogens	Patient Group		
	Pediatric (<18)	Adults (18–65)	Elderly (>65)
Number of positive patients/total number of patients	15/36 (41.67)	15/35 (42.85)	26/62 (41.9)
No. of positive PCR results	19	16	26
*Campylobacter* spp.	3	2	5
*Salmonella* spp.	4	1	3
STEC	0	2	0
*Shigella* spp.	0	1	0
*C. difficile*	3	3	14
Norovirus	5	5	4
Rotavirus	1	1	0
Astrovirus	2	1	0
Co-infections	3 ^a^	1 ^b^	0

^a^ *Campylobacter* spp., norovirus; norovirus, STEC; norovirus and astrovirus, *C. difficile.* ^b^ Astrovirus, *C. difficile.*

**Table 2:** Isolation of hospitalized patients.

	Median Time of Isolation (IQR), h		*p*-Value
	PCR-Negative (*n* = 50)	PCR-Positive (*n* = 35)	
Hospital length of stay	156.75 (114.33–261.51)	143 (112.5–259)	0.608
Single-room isolation	104 (22.75–156.75)	139.75 (67–245.5)	0.039

**Table 3:** *C. difficile* PCR-positive cases.

No.	Underlying Disease	Symptoms	Age	Sex	Time to PCR Result (h)	Length of Stay in the ED (h)	Hospitalization	CDI Diagnosis				Start of Antibiotic Treatment after Admission to the ED (h)	Iso-Lation (h)	Hospital Length of Stay (h)
								PCR Ct Value	WBC ^1^ (Gpt/mL)	Creatinine ^1^ (mmol/L)	Recurrence (R), First Diagnosis (F), or non Relevant (N)			
1	Stroke condition	Diarrhea and fever	74	f	4.83	11.5	No ^2^	26.3	24.8	88	R	Vancomycin and metronidazole (7.22)		
2	COPD ^3^, Crohn’s disease, recurrent UTI ^4^, and CHD ^5^	Diarrhea, kidney failure, and metabolic acidosis	73	m	4.25	6.77	Yes	24.7	12.6	246	F	Vancomycin (5.1) and piperacillin– tazobactam (5.75)	161.75	168.5
3	Alzheimer’s disease and recurrent UTI ^4^	Diarrhea	96	f	3.68	10.17	Yes	25.1	22.4	113	F	None	259	269.32
4	Diabetes	Diarrhea	93	f	4.14	N.D.	No ^2^	31.1	13.8	69	F	Metronidazole		
5	Kidney disease with dialysis and diabetes	Abdominal pain, nausea, and fever	78	m	12.5	4.48	Yes	24.3	8.1	349	R	None	254.5	259
6	Hirschsprung’s disease	Obstipation and vomiting	1	m	4.01	<1	Yes	27.7	14.3	20	N	None	47	47.15
7	Alcoholism	Diarrhea for one week and cachexia	69	f	3.63	11.67	Yes	20.9	5.6	60	F	Vancomycin (t.n.a. ^6^)	182.33	193.98
8	Rheumatoid arthritis and chronic renal failure	Diarrhea for one week and exsiccosis	94	m	15.3	2.93	Yes	21.9	24.1	184	R	Vancomycin (19.17)	331.25	334.17
9	Rectal carcinoma and chronic renal failure	Ileus, abdominal pain, and weight loss	83	m	13.75	4.1	Yes	31.7	15.4	106	N	Metronidazole (15.5)	0 ^7^	280.25
10	Ulcerative colitis and chronic renal failure	Diarrhea and fever	90	f	12.75	5.87	Yes	23.5	11.3	110	R	Vancomycin, metronidazole (9.5)	204	209.82
11	Granulomatosis, monoclonal gammopathy, diabetes, dementia, UTI ^4^, and sepsis	Diarrhea and fever	85	f	18	6.84	Yes	36.8	22.7	285	F	Vancomycin (t.n.a. ^6^) and piperacillin–tazobactam (2)	215.5	577.23
12	CHD ^5^, asthma, and osteosynthesis after femur fracture	Diarrhea for more than one week	85	f	19.6	5.22	Yes	28.3	15.1	132	F	Vancomycin (t.n.a. ^6^)	143	148.25
13	Chronic venous insufficiency, recurrent UTI ^4^, and abscessed symphysis	Diarrhea, exsiccosis, and fever	80	m	8.1	3.85	Yes	23.3	31	63	F	Vancomycin (t.n.a ^6^)	114.5	129.78
14	Stroke condition, adipositas, sepsis	Diarrhea and fever	75	f	22.6	4.25	Yes ^8^	22.2	21.9	80	F	Vancomycin. Metronidazole (t.n.a. ^6^) and piperacillin–tazobactam (0.7)	t.n.a. ^6^	t.n.a. ^6^
15	Malignant melanoma	Diarrhea and fever	46	m	12.43	2.75	Yes	26.3	4.9	39	F	Vancomycin and ampicillin–sulbactam (13.23)	417	420.03
16	Hirschsprung’s disease	Diarrhea and fever	<1	f	13.6	<1	Yes	23.2	18.4	N.D.	F	Metronidazole (14)	137.88	137.88
17	Pancreatic carcinoma	Diarrhea, exsiccosis, and abdominal pain	59	m	5.38	10.37	Yes	31.6	15	60	F	Vancomycin (15.25)	125.25	135.67
18	Alzheimer’s disease, CHD ^5^, diabetes, and chronic renal failure	Diarrhea	83	f	3.07	8.17	Yes	26	4.7	168	F	Vancomycin and metronidazole (6.1)	151.5	159.6
19	Hodgkin‘s disease, conditions after CDI, and sepsis	Diarrhea and vomiting	60	m	5.08	8.97	Yes	28.4	21.4	103	R	Vancomycin (8.75) and meropenem (4.75)	188.5	197.52
20	Short bowel syndrome, ileostomy	Abdominal pain, fever	12	m	5.5	4	No ^9^	29.4	6	41	N	None		

^1^ Reference ranges: white blood cells (WBC) 4.5–11.3 Gpt/L and creatinine 44–80 μmol/L. ^2^ Nursing home. ^3^ COPD: chronic obstructive pulmonary disease. ^4^ UTI: urinary tract infection. ^5^ CHD: coronary heart disease. ^6^ t.n.a.: time not available. ^7^ Hygiene management error. ^8^ Transfer into another hospital. ^9^ No clinical relevance.

## Data Availability

The dataset analyzed in this study is available from the corresponding author upon reasonable request.
